# External Validation of SHA_2_PE Score: A Score to Predict Low-Risk Lower Gastrointestinal Bleeding in the Emergency Department

**DOI:** 10.1155/grp/5657404

**Published:** 2025-01-03

**Authors:** Akram I. Ahmad, Ahmed El Sabagh, Jennie Zhang, Claire Caplan, Ahmad Al-Dwairy, Tarek Bakain, Faith Buchanan, Lea Fisher, Andrew Wilbur, Samantha Marshall, Garrett Buechner, Malaak Hamzeh, Rachna Dhanjal, Alexander Boos, Lynette Sequeira

**Affiliations:** ^1^Digestive Disease Institute, Cleveland Clinic Florida, Weston, Florida, USA; ^2^Internal Medicine Department, MedStar Washington Hospital Center, Washington, DC, USA; ^3^Gastroenterology Department, George Washington University, Washington, DC, USA; ^4^School of Medicine, Georgetown University, Washington, DC, USA

**Keywords:** hospitalization, low risk, lower gastrointestinal bleeding, score

## Abstract

**Introduction:** Lower gastrointestinal bleeding (LGIB) frequently leads to emergency department (ED) visits and hospitalizations, encompassing a spectrum of outcomes from spontaneous resolution to intrahospital mortality.

**Aim:** The purpose of this study was to validate a scoring system designed to identify cases of low-risk LGIB, allowing for safe discharge from the ED.

**Methods:** A retrospective analysis of all gastrointestinal bleeding cases presented at three EDs in 2020 was conducted, focusing specifically on patients with LGIB. The SHA_2_PE score incorporates factors such as systolic blood pressure, hemoglobin levels, use of antiplatelet or anticoagulant medications, pulse rate, and episodes of bright blood per rectum.

**Results:** Out of 1112 patients presenting with LGIB to the ED, 55 were hospitalized, 20 required blood transfusions, 15 underwent colonoscopies, one underwent interventional radiology procedures, and two patients died. Employing a SHA_2_PE score with a cutoff value of 1 yielded a specificity of 78.5% (95% CI (confidence interval) [75.8–81.0]), sensitivity of 76.8% (95% CI [63.6–87.0]), positive predictive value (PPV) of 17.0% (95% CI [12.6–22.2]), and negative predictive value (NPV) of 98.3% (95% CI [97.2–99.1]) for predicting the need for hospitalization and intrahospital intervention. When considering return visits to the ED within 7 days with the same presentation, the score demonstrated a specificity of 78.8% (95% CI [76.0–81.3]), sensitivity of 68.6% (95% CI [56.4–79.1]), PPV of 19% (95% CI [14.3–24.4]), and NPV of 97.2% (95% CI [95.8–98.2]).

**Conclusions:** The SHA_2_PE score demonstrates potential in predicting cases of low-risk LGIB, offering a high NPV for hospitalization, the need for intrahospital intervention, and return visits to the ED. However, these findings should be interpreted cautiously given the low prevalence of interventions and limitations in the study's population and design.

## 1. Introduction

Lower gastrointestinal bleeding (LGIB) is a prevalent reason for emergency department (ED) visits and subsequent hospital admissions within the United States. The annual incidence of LGIB in the United States has been reported to range from 35.7 to 41.8 cases per 100,000 population [1]. Notably, the burden of LGIB-related hospitalizations has exhibited a sustained increase over the past decade, coinciding with escalated utilization of healthcare resources [2].

In the context of ED presentations for LGIB without evidence of shock, the overall all-cause mortality rate stands at 2.7%. However, when LGIB is accompanied by shock upon presentation, this rate substantially escalates to 24.3% [3]. It is pertinent to acknowledge that more than 80% of LGIB cases will spontaneously cease without medical intervention; as a result, hospitalization and diagnostic procedures might not achieve a definitive diagnosis [4]. Hence, ED physicians face a challenge when evaluating patients with LGIB, leading to a need for careful consideration regarding whether admission is required.

Numerous clinical scoring systems have been created to predict the severity of LGIB and factors linked to adverse outcomes [5–9]. However, only one prior scoring system—Oakland score—was devised specifically for predicting safe discharge from the ED [10].

A novel scoring system called SHA_2_PE has been introduced to identify low-risk patients suitable for outpatient management. This score offers the advantage of simplicity and utilizes readily available clinical data such as systolic blood pressure, hemoglobin levels, the presence of anticoagulant or antiplatelet therapy, pulse rate, and event of bleeding in the ED. A score ≤ 1 indicates a low probability of requiring hospital admission. Another notable advantage of this scoring system is its high negative predictive value (NPV) of 96% and sensitivity of 91%, as demonstrated in the original dataset, enabling the identification of patients who can be safely discharged from the ED [11]. As to our knowledge, the SHA_2_PE score has not been externally validated in a North American cohort.

Our study is aimed at externally validating the SHA_2_PE score and assessing predictive accuracy using a patient population from MedStar health system EDs who presented with LGIB.

## 2. Methods

### 2.1. Study Design

This is a retrospective study conducted at MedStar Washington Hospital Center. The study was reviewed and approved by the MedStar Health Research Institute and Georgetown University Hospital Institutional Review Board. Transparent reporting of a multivariable prediction model for individual prognosis or diagnosis (TRIPOD) guidelines has been followed to report the results of our study [12].

### 2.2. Study Population

Patients were selected from three EDs in the Medstar health system. Convenient sampling was employed to obtain study population. Encounters were selected in 2020 using ICD-10 codes (K62.5, K57.21, K92.1, K92.2). Patients were included in the study if the presenting symptom was bright blood per rectum. Patients with evidence of upper gastrointestinal (GI) bleeds, hemorrhoids, or younger than 18 years old were excluded from the study.

### 2.3. SHA_2_PE Scoring

SHA_2_PE score includes systolic blood pressure less than 100 mmHg for 1 point, hemoglobin value (grams/deciliter) between 12 and 10.5 for 1 point and less than 10.5 for 2 points, using antiplatelet or anticoagulant, each for 1 point, having a pulse rate > 100 BPM for 1 point, and an episode of bright blood per rectum in the ED for 1 point; this is highlighted in Table 1.

Electronic medical records were reviewed to gather patients' information. We reviewed patients' hospitalization, demographics, and initial vital signs. Medication use, laboratory findings, intrahospital intervention, and mortality were examined. Intrahospital intervention includes blood transfusion, colonoscopy (including full colonoscopy and proctosigmoidoscopy), interventional radiology procedure, or surgical procedure during hospitalization. Each patient's record was reviewed by a single reviewer. We reviewed ED physician clinical notes to confirm inclusion and exclusion criteria. The lowest blood pressure, highest heart rate (HR), and lowest Hemoglobin (Hb) value were collected, if multiple values were available.

The study population was divided into two groups—intervention and no intervention, based on hospitalization, intrahospital intervention, and mortality. An additional analysis was done to evaluate the score predictive ability to predict patient who will return to the ED with the same presenting symptom within 7 days. Complete case analysis method was used to handle missing data.

### 2.4. Data Analysis

We presented the data using frequency and percentages for categorical variables, mean and standard deviation for continuous variables, and median and interquartile range (IQR) for highly skewed continuous variables. Comparison between groups was conducted using Fisher's exact test for categorical variables, a two-sample *t*-test for continuous variables, and Kruskal–Wallis test for highly skewed continuous variables.

Sensitivity, specificity, positive predictive value (PPV), and NPV were calculated for the SHA_2_PE score at a cutoff point of 1 (<= 1 vs. > 1) along with their confidence intervals (CIs) using the Clopper–Pearson method. The receiver operator characteristic (ROC) curve was plotted for the SHA_2_PE score, and the area under the curve (AUC) was obtained. Analysis was performed using R 4.0.0, R Foundation for Statistical Computing, Vienna, Austria.

## 3. Results

**Figure 1:**
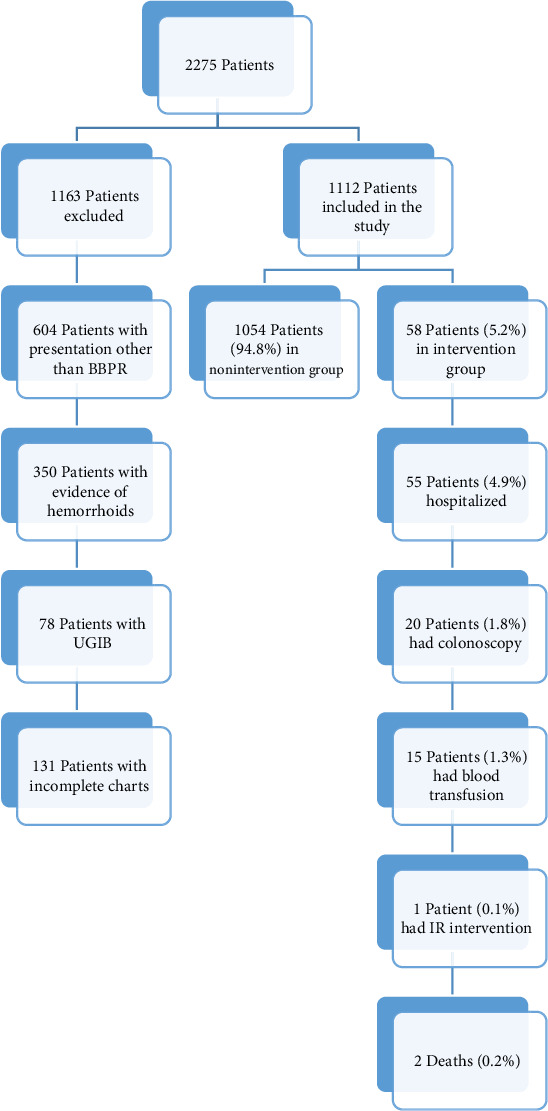
Study population and groups. BBPR: bright blood per rectum, UGIB: upper gastrointestinal bleed.

Two thousand two hundred and seventy-five patients were encountered in three EDs in 2020 with primary ICD-10 codes (K62.5, K57.21, K92.1, K92.2). Of those, 1112 patients were eligible for our study (Figure 1). The study population was divided into the intervention (58 patients) and nonintervention (1054 patients) groups. In the intervention group, 58 patients (5.2%) had positive study outcomes, 55 patients were hospitalized (4.9%), 20 patients had blood transfusions (1.8%), 15 patients had colonoscopy (1.3%), one patient had IR procedures (0.1%), and two deaths were reported in the study (0.2%). None of the patients required surgical intervention (Figure 1).

The mean age in the intervention group is 65.45 years compared to 48.29 years in the nonintervention group (*p* < 0.001). Females represent 52.6% (*n* = 585); African American, Caucasian, Hispanics, and other races\ethnicities consist 698 patients (63.2%), 302 patients (27.4%), 39 patients (3.7%), and 104 patients (9.4%) of the study population consecutively without significant difference between the study groups (Table 2).

The average systolic blood pressure, diastolic blood pressure, mean arterial pressure, and serum hemoglobin levels are significantly lower in the intervention group (*p* < 0.001). On the other hand, the mean pulse rate (*p* = 0.005), serum creatinine (*p* = 0.005), and blood urea nitrogen level (*p* < 0.001) are significantly lower in the nonintervention group (Table 1). Using anticoagulant, antiplatelet, or both was observed consecutively in 10 patients (17.2%), 8 patients (13.8%), and 2 patients (3.4%) in the intervention group and 56 patients (5.3%), 76 patients (7.2%), and 14 patients (1.3%) in the nonintervention group (*p* < 0.001).

**Figure 2:**
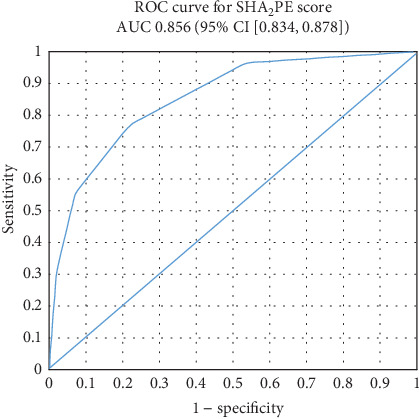
ROC curve based on the SHA_2_PE score.

One thousand and thirty-five patients have complete data for SHA_2_PE scoring, and 253 patients (24.4%) have a score of more than 1.  Table 3 highlights these results. We used a cutoff value of more than 1 for the SHA_2_PE score and study outcomes of hospitalization, blood transfusions, in-patient colonoscopy, IR procedures, surgical procedures, and deaths. Based on those, the SHA_2_PE score has a specificity of 78.5% (95% CI [75.8–81.0]), sensitivity of 76.8% (95% CI [63.6–87.0]), PPV of 17.0% (95% CI [12.6–22.2]), and NPV of 98.3% (95% CI [97.2–99.1]). The AUC for the ROC curve was 0.856 (95% CI [0.834, 0.878]) (Figure 2).

Fifteen patients (1.3%) of the study population returned to the ED within 7 days with bright blood per rectum. A subgroup analysis was done to include a return to the ED to the study outcome. When return to the ED was included, SHA_2_PE score has a specificity of 78.8% (95% CI [76.0–81.3]), sensitivity of 68.6% (95% [56.4–79.1]), PPV of 19% (95% CI [14.3–24.4]), and NPV of 97.2% (95% CI [95.8–98.2]).

## 4. Discussion

In our analysis of 1112 patients at MedStar Health ED, we established the SHA_2_PE score's effectiveness in reliably identifying individuals who could be safely discharged with a low risk of adverse outcomes. Notably, our study included patients based on their primary presentation with LGIB, irrespective of specific admission criteria. Furthermore, our investigation demonstrated the SHA_2_PE score's reliability in excluding patients at risk of returning to the ED due to recurrent bleeding episodes. The SHA_2_PE score effectively distinguished individuals eligible for safe discharge using five readily available clinical and laboratory criteria, obviating the need for specialized investigations, with NPV of 97.2%.

Prior investigations have reported LGIB mortality rates ranging from 3% to 15% [13–15], notably higher than our findings, which revealed a mortality rate of only 0.2% in our study cohort. It is worth noting that the higher mortality in previous studies may be attributed to their focus on hospitalized patients, who inherently carry a greater overall risk. In contrast, our study had a lower hospital admission rate of 5.2%, which diverges from other reports citing admission rates of up to 40% [16]. The exclusion of younger adults with hemorrhoidal bleeding in certain studies can explain this discrepancy. Common interventions in our patient population included hospitalization, colonoscopy, and blood transfusion, aligning with findings from prior reports [15, 17].

Our objective of validating the SHA_2_PE score in a US population revealed its effectiveness as a predictor of safe hospital discharge, with an AUROC (area under a receiver operating characteristic) of 0.86 and a notably high NPV of 97.2%, even when considering patients who returned to the ED within 7 days. This performance surpasses that of an external validation study conducted in Switzerland, where the AUROC was 0.77 and the NPV was 90% [18]. This difference can be explained by our larger study population. However, our cohort had a lower rate of interventions (5.2% compared to 20%), which represents a significant limitation in interpreting our results, as a low prevalence can enhance the NPV of the test. This necessitates cautious consideration when generalizing our findings to broader populations.

Implementing the SHA_2_PE score in the ED clinical workflow has the potential to reduce avoidable hospitalizations for LGIB. In light of the rising expenses related to hospital admissions and inpatient treatments (with a median cost of $14,756 for hospital admission) [19], the utilization of the SHA_2_PE score could reduce the healthcare costs associated with LGIB.

Multiple scores had been developed to identify patients with a high risk of mortality except for Oakland and Birmingham scores which were designed to assess safe discharge. Both had excluded patients who were already admitted to the hospital, and the Birmingham score excluded patients who were discharged from the ED. Additionally, both are heavily weighted by hemoglobin at presentation, and neither reported a high NPV as reported by the SHA_2_PE score [10, 20].

SHA_2_PE score was originally derived and validated using the Nordic European cohort [11]. However, in the present study, we showed that the SHA_2_PE score can be utilized in a US cohort with a high percentage of African Americans and Hispanics which increases the generalizability of the score [21].

Our study has recognizable limitations. First, the retrospective nature of the study limits the generalizability; however, the diverse and large cohort mitigates this limitation. Second, our study has the potential for selection bias, as the study population was based on specific ICD-10 codes and included only patients with complete data for analysis. This may have contributed to the lower rates of hospitalization, transfusion, and mortality observed. Additionally, high-risk cases may have been managed outside the ED setting, further influencing the observed outcomes. Third, the risk of having a patient with upper GI bleeding mislabeled as LGIB is mitigated in our study by excluding patients with any evidence of upper GI bleeding after the initial presentation. Another limitation is the inclusion of colonoscopy and hospitalization as interventions that were not included in the initial study. Moreover, our study might be limited by the fact that patients discharged from the ED were classified as not having received any intervention. This categorization may have impacted the overall interpretation of outcomes and should be considered when evaluating the findings. A larger prospective multicenter study might be beneficial to address these limitations.

## Figures and Tables

**Table 1:** SHA_2_PE score.

**SHA**_**2**_ ** PE score**	**Points**
Systolic blood pressure < 100 mmHg	1
Hemoglobin value (g/dL) between 12 and 10.5	1
Hemoglobin value (g/dL) < 10.5	2
Antiplatelet therapy	1
Anticoagulation therapy	1
Pulse rate > 100 BPM	1
Bright red bleeding per rectum in the ED	1

**Table 2:** Study population clinical characteristics.

	**Overall**	**Nonintervention group**	**Intervention group**	**p** ** value**
*N*	1112	1054	58	
Age (mean (SD))	49.19 (19.98)	48.29 (19.64)	65.45 (19.26)	< 0.001
Sex (%)				
Female	585 (52.6)	547 (51.9)	38 (65.5)	0.058
Male	527 (47.4)	507 (48.1)	20 (34.5)	
Race (%)				
AA	698 (63.2)	656 (62.7)	42 (73.7)	0.064
Other	104 (9.4)	103 (9.8)	1 (1.8)	
White	302 (27.4)	288 (27.5)	14 (24.6)	
Ethnicity (%)				
Hispanic	39 (3.7)	38 (3.8)	1 (1.8)	0.718
Non-Hispanic	1011 (96.3)	955 (96.2)	56 (98.2)	
Bleeding event in the ED (%)				
No	887 (79.8)	857 (81.3)	30 (51.7)	< 0.001
Yes	225 (20.2)	197 (18.7)	28 (48.3)	
MAP (mean (SD))	98.70 (19.55)	99.53 (19.04)	83.65 (22.53)	< 0.001
Systolic BP (mean (SD))	137.70 (23.73)	138.64 (23.10)	120.27 (28.26)	< 0.001
Diastolic BP (mean (SD))	81.64 (12.77)	82.28 (12.46)	69.82 (12.80)	< 0.001
Pulse (mean (SD))	85.13 (16.93)	84.63 (16.29)	94.09 (24.48)	0.005
Hg (mean (SD))	13.00 (2.15)	13.17 (1.91)	10.08 (3.49)	< 0.001
INR (median [IQR])	1.23 (0.91)	1.15 (0.52)	1.91 (2.24)	0.106
Anticoagulation use (%)				
No	1030 (92.6)	984 (93.4)	46 (79.3)	0.001
Yes	82 (7.4)	70 (6.6)	12 (20.7)	
Antiplatelet use (%)				
No	1012 (91.0)	964 (91.5)	48 (82.8)	0.033
Yes	100 (9.0)	90 (8.5)	10 (17.2)	
Anticoagulation and antiplatelet (%)				
None	946 (85.1)	908 (86.1)	38 (65.5)	< 0.001
Anticoagulant	66 (5.9)	56 (5.3)	10 (17.2)	
Antiplatelet	84 (7.6)	76 (7.2)	8 (13.8)	
Both	16 (1.4)	14 (1.3)	2 (3.4)	

Abbreviations: AA, African American; BP, blood pressure; ED, emergency department; Hg, hemoglobin; INR, international normalized ratio; MAP, mean arterial pressure; SD, standard deviation.

**Table 3:** Number of interventions according to the SHA_2_PE score.

	**Intervention**	**No intervention**	**Total**
SHA_2_PE > 1	58	195	253
SHA_2_PE ≤ 1	17	765	782
Total	75	960	1035

## Data Availability

The data used in this study are available upon reasonable request to the corresponding author. Access to data may be subject to institutional and ethical guidelines.
